# Essentials of Gynecology

**Published:** 1890-07

**Authors:** 


**Essentials of Gynecology,** arranged in the form of questions and answers prepared especially for the students of medicine. By EDWARD B. CRAGIN, M. D., Attending Gynecologist to the Roosevelt Hospital, out-patient department, etc. With 58 illustrations. Philadelphia: W. B. Saunders. 1890.

**Essentials of Diseases of the Skin,** including the Syphilodermata. Arranged in the form of questions and answers. Prepared expressly for students of medicine. By HENRY W. STELWAGON, M. D., Ph. D., Attending Physician to the Philadelphia Dispensary for Skin Diseases, etc. With 74 illustrations. Philadelphia: W. B. Saunders. 1890.

These two little books are numbers 10 and 11 of Students' Question Compends. They are handsomely printed and bound and serve the purpose for which they are attended as well as any we have seen. The uniform price of the series is 75 cents each, and can be obtained through the trade or from the publisher direct.

